# A Tool for Assessing the Experience of Shared Reality: Validation of the German SR-T

**DOI:** 10.3389/fpsyg.2019.00832

**Published:** 2019-04-16

**Authors:** Bjarne Schmalbach, Linda Hennemuth, Gerald Echterhoff

**Affiliations:** Department of Psychology, University of Münster, Münster, Germany

**Keywords:** shared reality, experienced commonality, common ground, scale development, communication, interpersonal relationships

## Abstract

Humans are highly motivated to achieve shared reality – common inner states (i.e., judgments, opinions, attitudes) with others about a target object. Scholarly interest in the phenomenon has been rapidly growing over the last decade, culminating in the development of a five-item self-report scale for Shared Reality about a Target (SR-T; Schmalbach, Rossignac-Milon, Keller, Higgins, and Echterhoff, unpublished). The present study aims to validate the German version of the scale. Individuals can establish shared reality either by receiving social verification (i.e., agreement or confirmation from an interaction partner) or by aligning their inner state with that of their partner. To increase the scope of the present validation, we implemented both pathways of shared-reality creation in three studies (*N* = 522). Study 1 employed a social judgment task, in which participants assessed ambiguous social situations and received confirming (vs. disconfirming) feedback from their partner. Studies 2 and 3 build on the saying-is-believing paradigm, in which participants align their own evaluation of the target with their partner’s judgment. Based on an evaluatively ambiguous description, participants communicated about a target person and later recalled information about the target (Study 2). To further generalize the findings, message production was omitted from the paradigm in Study 3. Overall, the five-item model of the SR-T evinced good fit and reliability. In Study 1, the SR-T reflected experimentally induced differences in commonality of judgments– even when controlling for several related state measures, such as Inclusion of Other in the Self and Need Threat. In Studies 2 and 3, the SR-T predicted participants’ evaluative recall bias, which is an established, indirect index of communicators’ shared-reality creation. This effect was stronger when participants overtly communicated with their study partner, but it still emerged without overt communication. Across all studies, correlations with related constructs support the convergent validity of the SR-T. In sum, we recommend the use of the SR-T in research on interpersonal processes and communication.

## Introduction

Humans are motivated to create shared realities with other (Echterhoff et al., 2009a; Echterhoff and Higgins, 2017; Higgins, 2019), often through interpersonal communication (Echterhoff and Schmalbach, 2018). For example, when people meet a new employee at their workplace, they tend to form their impressions of the newcomer jointly with their colleagues, and they feel more confident in their impressions when others agree. Shared reality creation allows us to evaluate other people (e.g., Echterhoff et al., 2005, 2008) or groups (Conley et al., 2016); to form culturally shared knowledge (Lau et al., 2001; Kashima et al., 2018) as well as political opinions (Jost et al., 2008; Stern and Ondish, 2018) and moral convictions (Heiphetz, 2018). Mastering successive stages of shared reality competence has been described as a key principle of child development (Higgins, 2016; Liszkowski, 2018). Furthermore, repeated experiences of shared reality lay the groundwork for relationships (Andersen and Przybylinski, 2018; Rossignac-Milon and Higgins, 2018) and can help us to develop and maintain a sense of who we are and what we want (Sullivan, 1953; Higgins, 1996). Shared reality has even been conceptualized as a gateway to ideological radicalization (Kruglanski et al., 2014). In addition to its epistemic functions of helping individuals in forming reliable impressions (Echterhoff and Higgins, 2017), shared-reality creation comes with another immense benefit for members of an “ultrasocial” species (Tomasello, 2014): When we create a shared reality with others, we connect with them, we establish or strengthen our social relationships, and thus fulfill our fundamental need for belonging (Baumeister and Leary, 1995).

For a valid and reliable assessment of the construct of shared reality, a standardized measure is needed. Previous research into shared reality has often employed the saying-is-believing paradigm (Higgins and Rholes, 1978) and used an evaluative recall bias as a proxy for shared reality. After communicating with an audience about a target (who is familiar to the partner), participants’ recall of the original ambiguous information about the target is distorted toward the audience’s judgment of the target. That is, when the audience likes (vs. dislikes) the target, participants remember it in a more positive (vs. negative) way (see section “*Procedure*” of Study 2 for a more detailed description). According to a shared reality explanation, the strength of the evaluative recall bias reflects the strength of the shared reality participants established with their audience. However, in practice there are several limitations with this measure. Apart from its reliance on trained coders and a time-consuming procedure, its most striking caveat is that it is tied to this specific research design – talking with someone about a target that the participant is previously unfamiliar with. Furthermore, existing *ad hoc* self-report measures related to shared reality have included various aspects other than the defining aspect of shared reality, that is, the experienced commonality of inner states with a partner (e.g., Echterhoff et al., 2008; Bratanova and Kashima, 2014).

To remedy these shortcomings, Schmalbach et al. (unpublished) recently developed a state measure of shared reality regarding a current target (SR-T). To achieve this, they compiled all items in the extant literature and validated the SR-T in five studies, demonstrating its reliability and validity. Most importantly, the SR-T mediated the relationship between a first instance of shared reality –experimentally induced by high (vs. low) agreement, or verification, from the partner – and a second instance, in which participants had the opportunity to align their judgments with those made by their study partner. The SR-T scale is designed to provide a precise assessment of the core of shared reality – the experience of common inner states.

To make the SR-T accessible to the German-speaking research community and to allow for culturally specific research, we translated the instrument and aim to validate it in the present set of studies. To this end, we employ three paradigms that represent potential research designs in which the SR-T could be usefully applied. To obtain a German version of the scale, two translators converted the English items into German ones independently of one another and back-translated them. Afterward, their results were compared and potential differences were discussed until a consensus was found. In Study 1, we examined basic psychometric properties – item and scale descriptive statistics and factor loadings in exploratory factor analysis (EFA) – to assess the quality of the translated items and reduce the item pool. Additionally, we tested the SR-T’s diagnostic validity – that is, its capability to assess and predict different degrees of shared reality, which we experimentally induced by creating conditions of high (vs. low) commonality of judgments. Studies 2 and 3 primarily served the purpose of analyzing the German SR-T’s factorial validity in confirmatory factor analysis (CFA). Moreover, we extended the work by Schmalbach et al. (unpublished) by testing whether the SR-T – as a measure of common inner states – can predict participants’ evaluative recall bias. Across all three studies, we also examined convergent and discriminant validity by checking correlations with various measures of interest.

## Study 1

Study 1 was conducted at a time when the construction of the English version of the SR-T was not completed. Thus, we based our first experiment on a preliminary item pool and examined the factor structure for the German translations. Moreover, we examined whether the extent of objective agreement or commonality (*high* vs. *low*) with the partner’s judgment is reflected in a preliminary German SR-T. The manipulation has been successfully employed to induce experiences of high vs. low commonality Schmalbach et al. (unpublished).

The previous considerations imply that the experience of shared reality is context-sensitive. This context-sensitivity is also reflected by intergroup biases in shared-reality creation (Echterhoff et al., 2005, 2008, 2017). In several studies, participants tuned their *messages* to the audience’s attitude regardless of whether the audience belonged to the participants’ ingroup or to an outgroup. However, no evidence for a shared-reality creation with the outgroup audience was found for the audience-tuning *memory* bias. When the audience was an ingroup member (e.g., when German communicators talked to a German audience), the audience-tuning memory bias was found. In stark contrast, no such memory effect was found when the audience was an outgroup member (e.g., when German communicators talked to a Turkish audience).

Building on these findings, we tested whether the group identity of the communication partner (*ingroup* vs. *outgroup*) affected the experience of commonality. Furthermore, we explored whether the experience of shared reality with an outgroup audience may improve participants’ attitudes toward the outgroup in general.

In terms of convergent validity, we expected high correlations of the SR-T with participants’ epistemic and relational assessments of their communication partner (Chun et al., 2017). On a related note, Inclusion of Other in the Self has been shown to have substantial relationship to the SR-T in previous research (Schmalbach et al., unpublished). Interpersonal connectedness has been theorized as a main outcome of experiencing a shared reality (Hardin and Higgins, 1996). Similar correlations were to be expected for liking (Festinger et al., 1952). Furthermore, we hypothesized SR-T to correlate positively with euthymia and negatively with arousal and anxiety because of shared reality’s function in satisfying existential needs (Baumeister and Leary, 1995). Accordingly, we also assumed that participants that share reality with their study partner will exhibit a higher self-esteem. Because, social curiosity captures interest in social information and contact (Renner, 2006), it should correlate positively with SR-T. We expected tolerance of interpersonal ambiguity to be negatively associated because individuals with low tolerance should be more motivated to reach an unambiguous view of the situation. The same is true for participants with high consensus motivation. Consensus motivation represents the negative dimension of argumentativeness (Infante and Rancer, 1982). As such, it is associated with a need to minimize discussion and reach a conclusion. In a similar line of argument, we expected the social orientation – common with experiencing a shared reality – to be associated with tendencies of social desirability.

### Materials and Methods

#### Participants

For Study 1, we aimed for a sample of at least 200 participants – a common recommendation in factor analysis research which also coincides with the 10:1 observations-to-indicators rule (Arrindell and van der Ende, 1985; MacCallum et al., 1999). Additionally, a sample size of at least *n* = 171 is needed to find a moderately sized effect of *d* = 0.50, given α = 0.05 and β = 0.10. We recruited a sample of *n* = 218 participants through distributing the study link on online platforms such as Facebook groups and other bulletin boards. Age was collected in response bins: Most participants were 29 years old or younger (58.3%), and only 11% were older than 50 (*Min* = 18; *Max* = 78). We approximated the mean by taking the scale midpoints, yielding a mean age of 32.36 (*SD* = 12.73). Gender was not distributed evenly (*n*_female_ = 133, 61.0%; *n*_male_ = 83, 38.1%; *n*_other_ = 2, 0.9%). The majority of the sample were students (*n* = 99, 45.4%) or employed for wages (*n* = 73, 33.5%). Furthermore, participants were relatively well-educated, a majority of them having achieved a degree (*n* = 128, 58.7%). No participants were excluded after analyzing the suspicion checks. As a compensation for taking part in the study, participants were given the opportunity to enter a lottery for three 20€ book vouchers.

#### Procedure

Upon declaring their informed consent, participants had to give themselves a nickname for the purpose of the study. They were then informed that they would be working together with “Khaled” or “Michael,” respectively. We employed a social judgment task using the multi-motive grid (MMG; Schmalt et al., 2000; Sokolowski et al., 2000), a diagnostic instrument modeled after the Thematic Apperception Test. Ostensibly working together with a second participant, individuals had to first make up their own mind about what the pictures were depicting: They were presented with four short sentences, e.g., “The man with the ball feels alone.” – possible interpretations of the depicted scene – and had to rate their agreement with those statement on a scale of 1 “don’t agree at all” to 6 “completely agree.” Afterward, they received feedback as to how their study partner rated the statements. We manipulated the feedback, making the partner’s ratings either close to the ones the participant gave (*High Commonality condition*) or more distant (*Low Commonality condition*). This method was previously applied successfully in work by Niemeier (2011). By pairing up participants with a partner who has a typically *German* (“Michael”) or typically *Middle Eastern* name (“Khaled”) we additionally manipulated whether participants will likely perceive their partner as an in- or outgroup member. After completing this procedure for five pictures, participants filled out the SR-T and additional questionnaires, and gave their sociodemographic information.

#### Measures

The SR-T item pool in Study 1 included those seven items presented in Table 1 (see Appendix for translations). Participants rated their experience of a shared reality on seven-point scales, ranging from “strongly disagree” to “strongly agree.” Additionally, they replied to the Epistemic Trust (ω = 0.946) and Relational Motivation (ω = 0.818) scales, consisting of three items each (Schmalbach et al., unpublished; see Table 1 and Appendix B for item phrasing). To assess participants’ attitudes toward the outgroup we used a six-item measure (ω = 0.834; Gümüs et al., 2014). The social curiosity scale (SCS; ω = 0.905; Renner, 2006) measures a person’s interest in their social surroundings. We employed the general social curiosity subscale, consisting of five items such as “When I meet a new person, I am interested in learning more about him/her.” Answer options reach from 1 “strongly disagree” to 4 “strongly agree.” Tolerance of interpersonal ambiguity (TIA; ω = 0.904; Wolfradt and Rademacher, 1999) was assessed using a 10-item measure of interpersonal and emotional aspects of tolerance of ambiguity. Response options range between 1 “not true at all” and 7 “completely true.” Consensus Motivation (Blickle et al., 2000) was measured using two scales dealing with the individual‘s drive to look for agreement based on the topic (five items; ω = 0.807) and based on the person (four items; ω = 0.679). We used the arousal (five items; ω = 0.870), anxiety (five items; ω = 0.878), and euthymia (five items; ω = 0.914) subscales of the state trait anxiety depression inventory (STADI; Laux et al., 2013) to assess positive and negative moods. Inclusion of other in the self (IOS; Aron et al., 1992) is a one-item measure of interpersonal connectedness.

**Table 1:** Descriptive statistics and exploratory factor analysis of the initial item pool (Study 1).

	*M*	*SD*	γ_1_	γ_2_	*r_it_*	F1	F2
SR-T 1: X und ich haben die gleichen gedanken und gefühle über Y.	3.271	1.897	0.210	-1.436	0.955	0.995	-0.060
SR-T 2: Ich stimme mit Xs einstellungen in bezug auf Y überein.	3.353	1.760	0.126	-1.318	0.927	0.918	0.028
SR-T 3: Meinem empfinden nach steht meine sichtweise mit der von X in einklang.	3.257	1.873	0.237	-1.382	0.961	0.952	0.016
SR-T 4: Ich denke, dass X und ich bezüglich Y auf derselben wellenlänge liegen.	3.266	1.906	0.214	-1.416	0.964	0.976	-0.018
SR-T 5: Ich empfinde ähnlich wie X in bezug auf Y.	3.294	1.880	0.261	-1.319	0.956	0.990	-0.047
SR-T 6: Ich stimme mit Xs standpunkt in bezug auf Y überein.	3.266	1.835	0.198	-1.392	0.948	0.978	-0.045
SR-T 7: X und ich sehen Y gleich.	2.963	1.831	0.423	-1.182	0.908	0.935	-0.040
ET 1: Man kann sich auf X eindruck von den bildern verlassen.	3.633	1.710	-0.067	-1.125	0.878	0.615	0.365
ET 2: X ist eine glaubwürdige informationsquelle in bezug auf Y.	3.610	1.714	-0.004	-1.163	0.950	0.551	0.463
ET 3: X ist jemand, dessen urteil über Y man vertrauen kann.	3.610	1.696	0.049	-1.098	0.919	0.525	0.487
RM 1: Ich möchte mich gut verstehen mit X.	3.812	1.594	-0.136	-0.537	0.751	-0.059	0.849
RM 2: Ich denke, dass X ein sympathischer mensch ist.	4.225	1.395	-0.274	0.000	0.626	0.145	0.664
RM 3: Ich würde gerne mehr zeit mit X verbringen.	3.156	1.510	0.129	-0.683	0.699	-0.059	0.776

*γ_*1*_, skewness; γ_*2*_, excessive kurtosis; *r_*it*_*, corrected item-total correlation with regard to the relevant subscale; “SR-T” means that we expected this item to load on a “perceived commonality” factor; “ET” means “epistemic trust,” and “RM” means “relational motivation.”*

Participants choose between seven progressively further overlapping pairs of circles to represent their relation to the study partner. Aron et al. (1992) report the retest reliability as *r* = 0.83. To assess self-esteem, we used the state self-esteem scale (SSES; Rudolph et al., 2008). We used the Performance (ω = 0.832) and Social (ω = 0.863) subscales, which consist of five items each. We employed the Liking subscale of the Rubin Scales (ω = 0.932; Amelang, 1991), which includes four items to assess interpersonal liking and perceived similarity. The Münster epistemic trust inventory (METI; ω = 0.974; Hendriks et al., 2015) is an instrument that measures epistemic trust in a source, using 14 items of paired adjectives. Finally, we utilized the social desirability short scale (KSE-G; Kemper et al., 2012) to capture response tendencies. It consists of two scales with three items each: Exaggeration of positive qualities (PQ+; ω = 0.677) and minimization of negative qualities (NQ-; ω = 0.616).

#### Analysis Plan

For the EFA, we used three methods: First, we employed parallel analysis (PA; Horn, 1965; Hayton et al., 2004) and the minimum average partial test (MAP; Velicer, 1976) to identify the ideal number of components. PA extracts eigenvalues based on random correlation matrices which are parallel to the empirical data. They were then compared for significant differences. The MAP test, on the other hand, presents that number of factors as optimal that minimizes the average partial correlations between items. O’Connor (2000) provides a syntax for both methods. Second, we ran principal axis factoring (PAF) with Oblimin rotation. As per recommendations from Trizano-Hermosilla and Alvarado (2016), we report McDonald’s ω (McDonald, 1999) as a measure of internal consistency. Furthermore, we calculated ANOVAs with η_p_^2^ and 90% confidence intervals. Moderation analyses were conducted in R using the *psych* package (Revelle, 2018).

### Results

#### Descriptive Statistics

We report item descriptive statistics in Table 1. All item means were situated around the middle point of the scale, indicating desirable item difficulty. We used Shapiro-Wilk tests to test for deviation from the normal distribution. They were all highly significant (*p* < 0.001), indicating non-normal distributions. In contrast, absolute skewness and excessive kurtosis were well within the common cut-off criteria of two and four, respectively (West et al., 1995), reflecting normal distribution.

#### Factor Structure

Parallel analysis presented clear evidence for two factors based on the empirical eigenvalues, while the MAP test was more ambiguous, indicating both, a two-factorial and a three-factorial solution (see Table 2). The PAF loadings (see Table 1) can be taken as further evidence for three distinct components: The items we expected to reflect experienced commonality (SR-T 1 to SR-T 7) loaded strongly on the first factor, which in turn made up the majority of all variance in all tested items. Relational motivation (RM 1 to RM 3) loaded primarily on the second factor with small cross-loadings. The epistemic trust items (ET 1 to ET 3), however, exhibited moderate factor loadings on both components. This particular loading pattern demonstrates how the three hypothesized constructs (experienced commonality, epistemic trust, and relational motivation) can be distinguished from one another based on data.

**Table 2:** Results for the exploratory factor analysis (Study 1).

	Minimum average		
	partial test		Parallel analysis			
# of			Raw	% of
factors	Squared	Power 4	data	variance	Means	95% *CI*
0	0.4970	0.3163				
1	0.1548	0.0337	9.339	71.842	1.425	1.528
2	0.0638	0.0097	1.613	12.406	1.317	1.386
3	0.0485	0.0105	0.596	4.581	1.235	1.295
4	0.0691	0.0333	0.425	3.269	1.163	1.215
7	…	…	…	…	…	…

*The lowest average partial correlation signifies the optimal solution, according to the MAP test. Eigenvalues that are higher than the 95% *CI* of the randomly generated eigenvalues should be interpreted as optimal.*

#### Validity

To examine diagnostic validity, we conducted a 2 (*commonality*) × 2 (*group membership*) ANOVA for SR-T. For this, we used a preliminary SR-T scale, consisting of Items SR-T 4 to 7 (ω = 0.975), which correspond with four of the five items in the English SR-T. We found a substantial main effect for commonality condition, *F*_(1,214)_ = 636.425, *p* < 0.001, η^2^*_p_* = 0.748 [0.701; 0.780]. This is strong evidence for the SR-T’s capability of capturing and differentiating between shared realities of various intensities. As can be seen in Table 3, the low commonality group exhibited markedly lower values than the high commonality group, several standard deviations apart from one another, *d* = 3.453 [3.031; 3.871]. There was no effect for group membership condition, *F*_(1,214)_ = 0.045, *p* = 0.832, η^2^*_p_* = 0.000 [0.000; 0.011], and no interaction effect, *F*_(1,214)_ = 0.501, *p* = 0.489, η^2^*_p_* = 0.002 [0.000; 0.025]. This is evidence that participants had the same experience of commonality, regardless of whether they assumed they were communicating with an ingroup or an outgroup member. When controlling for the state measures that were included in the study (such as IOS, METI, and others), the main effect of commonality condition on the SR-T remained significant, explaining roughly half of the existing variance (see Table 4). This is evidence, that the SR-T in fact captures a facet of interaction previously untapped by other measures. Neither commonality condition *F*_(1,212)_ = 2.555, *p* = 0.111, η^2^*_p_* = 0.012 [0.000; 0.047], nor group membership, *F*_(1,212)_ = 0.641, *p* = 0.424, η^2^*_p_* = 0.003 [0.000; 0.027], had a significant impact on general outgroup attitude. Neither did the interaction between the two, *F*_(1,212)_ = 0.397, *p* = 0.529, η^2^*_p_* = 0.002 [0.000; 0.023]. The SR-T was not associated significantly with outgroup attitude in either of the group membership conditions: *r*_Khaled_(107) = 0.064, *p* = 0.507, *r*_Michael_(105) = -0.138, *p* = 0.157. Next, we examined convergent validity (Table 5). As expected, SR-T correlated highly with epistemic and relational outcomes. Both, the Epistemic Trust scale of our own design and the METI scale evinced correlations of around 0.50 and higher. The same is true for the relational motivation scale, the Liking scale, and IOS. There were small associations with consensus motivation with regard to the person, euthymia, and minimizing of negative qualities. None of the other correlations were significant.

**Table 3:** SR-T, epistemic trust, and relational motivation by conditions (Study 1).

	Ingroup partner			Outrgroup partner		
	*M*	*SD*	*n*	*M*	*SD*	*n*
**SR-T**						
High commonality	4.736	1.042	55	4.675	1.150	57
Low commonality	1.547	0.707	53	1.660	0.580	53
**epistemic trust**						
High commonality	4.794	0.967	55	4.632	1.356	57
Low commonality	2.453	1.261	53	2.472	1.187	53
**relational motivation**						
High commonality	3.964	1.205	55	4.275	1.228	57
Low commonality	3.170	1.272	53	3.465	1.225	53

**Table 4:** ANCOVA for SR-T by commonality manipulation controlling for METI, IOS, liking, arousal, anxiety, and euthymia (Study 1).

	*F*	*p*	η^2^*_p_*
Commonality manipulation	153.708	<0.001	0.426
METI	0.596	0.441	0.003
IOS	43.732	<0.001	0.174
Liking	14.031	<0.001	0.063
STADI arousal	0.019	0.891	0.000
STADI anxiety	0.770	0.381	0.004
STADI euthymia	2.490	0.116	0.012
SSES performance	12.634	<0.001	0.058
SSES social	0.201	0.654	0.001

*METI, Münster epistemic trustworthiness inventory; IOS, inclusion of other in the self; STADI, stait-trait-anxiety-depression-inventory; SSES, state self-esteem scale.*

**Table 5:** Correlation matrix (Study 1).

	1	2	3	4	5	6	7	8	9	10	11	12	13	14	15	16	17	18
1 – SR-T	–	0.799***	0.493***	-0.034	0.794***	0.125	0.023	0.717***	0.107	0.076	-0.034	0.169*	0.044	-0.071	0.220**	-0.469***	0.023	0.142*
2 – Epistemic trust		–	0.649***	-0.012	0.678***	0.111	0.024	0.812***	0.085	0.077	-0.032	0.192**	0.084	0.001	0.196**	-0.562***	0.108	0.060
3 – Relational motivation			–	0.118	0.455***	0.231**	0.019	0.701***	0.047	0.083	0.027	0.110	0.063	0.018	0.218**	-0.463***	0.176**	-0.074
4 – Outgroup attitude				–	-0.019	0.245***	-0.011	-0.001	-0.142*	0.007	0.336***	-0.143*	-0.173*	-0.099	0.077	-0.130	0.196**	-0.257***
5 – IOS					–	0.138*	0.021	0.633***	-0.029	0.033	0.041	0.068	0.083	-0.089	0.211**	-0.393***	0.021	0.015
6 – Social curiosity						–	0.146*	0.211**	-0.067	0.096	0.350***	0.074	0.068	0.077	0.263***	-0.112	0.308**	0.016
7 – Tolerance of ambiguity							–	0.035	0.324***	0.539***	-0.089	0.253***	0.293***	0.331***	0.147*	0.210**	0.065	0.088
8 – Liking								–	0.064	0.079	0.034	0.177**	0.098	-0.017	0.244***	-0.581***	0.050	0.088
9 – SSES (performance)									–	0.571***	-0.107	0.178**	0.353***	0.422***	-0.158*	0.280***	-0.009	0.216**
10 – SSES (social)										–	-0.006	0.296***	0.379***	0.458***	-0.117	0.129	-0.004	0.115
11 – CM (topic)											–	-0.180**	-0.098	-0.009	0.131	-0.050	0.338***	-0.265***
12 – CM (person)												–	0.245***	0.182**	0.093	0.009	0.033	0.193**
13 – STADI arousal													–	0.609***	0.049	0.181**	-0.053	0.172*
14 – STADI anxiety														–	0.110	0.156*	0.015	0.092
15 – STADI euthymia															–	-0.188**	0.134*	-0.009
16 – METI																–	-0.022	0.113
17 – PQ+																	–	-0.295***
18 – NQ-																		–

*^∗^Significant at the 0.05 level; ^∗∗^Significant at the 0.01 level; ^∗∗∗^Significant at the 0.001 level. SR-T, shared reality about a target; IOS, inclusion of other in the self; SSES, state self-esteem scale; CM, consensus motivation; STADI, stait-trait-anxiety-depression-inventory; METI, Münster epistemic trustworthiness inventory; PQ+, exaggeration of positive qualities; NQ-, minimization of negative qualities.*

### Discussion

Study 1 served to narrow down the item pool via EFA and to find evidence for the translated SR-T’s validity. This was accomplished as the SR-T captured a majority of variance created by the *commonality* manipulation, presenting evidence for its diagnostic validity. This was true even when controlling for a number of established measures – such as the IOS -, indicating the SR-T’s incremental validity above and beyond those. There were no differences in the extent to which participants created a shared reality based on partners of different group membership. However, experiencing a shared reality – even with an outgroup member – did very little to improve participants’ attitude toward this group in general.

It should again be noted that Study 1 was conducted at a time when the construction of the English SR-T was not fully completed. Therefore, we were unable to include all items of the final version and chose an exploratory approach for clarifying the factor structure. In addition, we used a preliminary version of the scale for the rest of the analyses.

We suspect that the outgroup intervention – a small number of relatively inconsequential shared realities with a supposed outgroup member – may not have been sufficiently strong to induce lasting changes or even generalize to the outgroup as a whole. Participants scored similarly for SR-T, epistemic trust, and relational motivation with an outgroup partner as they did with an ingroup partner, indicating an equivalent in-the-moment experience. There was even a marginally significant trend for higher relational motivation for participants in the outgroup condition. However, this positive experience did not influence participants’ general attitude toward the outgroup. This could also be due to our sample being relatively young, educated, and – presumably – left-leaning. Their views of outgroup members may already be relatively egalitarian. Future studies interested in the prejudice-reducing nature of shared reality should take this into account.

## Studies 2 and 3

Studies 2 and 3 build on the saying-is-believing paradigm (Higgins and Rholes, 1978). Study 2 in particular closely followed the original design, which is described in more detail in the *Procedure* section. Traditionally, participants’ tuning of their recall – the extent to which their memory exhibits and evaluative bias reflecting their partner’s views – has been used as a quantified measure of shared reality. Accordingly, we expected that the SR-T predicts recall tuning. Experiencing a shared reality satisfies fundamental human needs. Therefore, we expected to replicate the negative correlations between the SR-T and Need Threat (Schmalbach et al., unpublished). Shared Identity refers to interpersonal connectedness on a group level. As such, we had similar expectation as for IOS. Need for cognitive closure (NFCC; Pierro et al., 2003) has previously been shown to be related to shared reality creation (Echterhoff et al., 2009b) higher need for closure should lead to stronger dependence on the study partner. Consequently, we expected positive associations with the SR-T.

In contrast, for Study 3, we omitted message production. Here, we investigated individual’s readiness to integrate information from a (*more* or *less*) credible source under *high* (or *low*) epistemic uncertainty. According to the epistemic process framework (Echterhoff and Higgins, 2017), individuals rely on these two epistemic inputs to judge the validity of information. Additionally, we expected that the SR-T would be associated with perceived generalized similarity. That is, experiences of shared reality may enhance the sense of perceived similarity with the communication partner (see Schmalbach et al., unpublished). Finally, we assessed Shared Reality-Need, which is a hypothesized personality disposition that leads individuals to more actively seek out shared realities in general. They are more dependent on others to validate their views and to perceive instances of consensus.

### Materials and Methods

#### Participants

To detect the main effect of interest in Study 2 – the correlation between SR-T and recall tuning – we needed to collect a sample of at least *n* = 109, given α = 0.05, β = 0.10, and *r* = 0.30. In addition, we ran simulation studies for the CFA using *simsem* (Jorgensen et al., 2018a), which started approaching 95% coverage around sample sizes of *n* = 100 given a one-factor model with five indicators and λ = 0.800 (cf. Wolf et al., 2013). To account for some shrinkage, we collected 120 participants on the campus of the University of Münster. Of those 120 who originally gave their consent, we excluded eight from the analysis because they either did not complete the recall measure or expressed precise suspicions about the purpose of the study. The average age of the sample of *n* = 112 was 23.21 (*SD* = 5.16; *Min* = 18; *Max* = 55) and there were roughly the same number of female and male participants (*n*_female_ = 60, 53.6%; *n*_male_ = 52, 46.4%). For taking part in the study, participants received either course credit or monetary compensation.

For Study 3, participants were again recruited via online message boards in addition to student bulletin boards. We strove for a sample of 215 participants, based on a moderate effect size of *r* = 0.20, α = 0.05, and β = 0.10, accounting for some shrinkage. Of the 215 participants originally collected, 23 had to be excluded because they did not complete the recall or expressed suspicions about the purpose of the study. The remaining sample of *n* = 192 had a mean age of 30.47 (*SD* = 10.47; *Min* = 18; *Max* = 73) and gender was distributed about equally (*n*_female_ = 102, 52.8%; *n*_male_ = 91, 47.2%). After completing the study, participants had the opportunity to participate in a lottery.

#### Procedure

In Study 2, after being informed of the general purpose of the study, participants gave their consent to partake. During the experiment, they worked on what is generally known as the saying-is-believing paradigm (Higgins and Rholes, 1978): in a supposed computer-based communication with a research group volunteer (“Jan”), they were given the task to describe a second volunteer (“Michael”). They were told that Jan knows Michael from the research group and that he *likes* (vs. *dislikes*) him. Participants read an ambivalent text describing Michael as a basis for their description (for material see Echterhoff et al., 2008). After writing the descriptive message and sending it to Jan, they worked on an unrelated filler task for around 5 min, to give Jan time to read the description. They were then informed, whether Jan was able to identify Michael *successfully* (vs. *failed* identification), before they had to recall the information describing Michael the information they had received originally. Before the end of the experiment, they answered the SR-T and additional scales, also giving basic sociodemographic information. Two independent coders rated participants’ messages and recalls in the manner described in Echterhoff et al. (2005): Each text unit is assigned a value between -5 and +5 to represent its global valence. After controlling the correlations for sufficient rater overlap (*r*_Message_ = 0.877; *r*_Recall_ = 0.892, both correlations *p* < 0.001), we took the mean of both ratings, yielding Message Valence and Recall Valence. By selectively inverting the scores for just those participants in the dislike condition while leaving those in the positive condition unchanged, we obtained Message Tuning and Recall Tuning – measures of the extent to which the audience attitude was incorporated, irrespective whether it was positive or negative.

In Study 3, after giving their informed consent and sociodemographic information, participants first completed a social judgment task, which we used to manipulate their epistemic certainty and needs (see Kopietz et al., 2010, for a more detailed description). Participants received (*positive, negative*, or *no*) feedback regarding their performance in evaluating ambiguous social settings correctly, putting them in either a *high need*, a *low need*, or a control condition. The feedback was counterbalanced with (*negative, positive*, or again *no*) feedback in a non-social cognitive task, to avoid mood effects. Participants were then told that they would read a volunteer’s (“Phillip’s”) thoughts about a fellow member of a research group (“Michael”), whom he has known *for several years* (vs. just got to know him *very recently*). The materials used for this can be accessed in the Appendix. We thus manipulated Phillip’s *credibility* as an information source, while keeping his attitude toward the target constantly positive. After reading the information, participants worked on an unrelated filler task for approximately 5 min and were then asked to recall the original information. Their recalls were coded in the same fashion as in Study 2 (*r*_Recall_ = 0.919, *p* < 0.001). Afterward, they answered several scales.

#### Measures

The SR-T, Epistemic Trust (ω = 0.896), and Relational Motivation (ω = 0.781) scales used in Study 2 were largely identical to the ones in Study 1. Merely one item (“I agree with X’s perception of Y”). We expect it to fit in well with the rest of the SR-T scale as it is a translation of the fifth item of the final English SR-T (Schmalbach et al., unpublished). To evaluate existential needs, we used a translation of the Need Threat Scale (NTS; 12 items; Gerber et al., 2017). It uses three items each to assess threats to an individuals’ belonging (ω = 0.468), control (ω = 0.717), meaningful existence (ω = 0.788), and self-esteem (ω = 0.534). Additionally, we employed the need for cognitive closure scale (NFCC; von Collani, 2014) to assess participants’ tendency to seek definitive conclusions and clear answers. It consists of three subscales: preference for structure and predictability (PSP; 18 items; ω = 0.903); personal need for structure (PNS; 10 items; ω = 0.900), and Undecidedness (seven items; ω = 0.857). Shared identity (SI; five items; ω = 0.854; Tanis and Postmes, 2008) refers to the extent to which a participant experiences themselves as having a common identity or group membership with their communication partner (Jan). Finally, we again included IOS as a measure of interpersonal connectedness.

In Study 3, we used the same items as in Study 2 to assess experienced commonality (SR-T), Epistemic Trust (ω = 0.907), and Relational Motivation (ω = 0.840). Similarly, we again employed the IOS as a brief assessment tool of interpersonal connectedness. Additionally, we measured Perceived Generalized Similarity (five items; ω = 0.947; Rossignac-Milon et al., unpublished). Participants rated on a scale of 1 “Strongly disagree” to 7 “Strongly agree” to what extent they agree that “My partner and I are very similar people.” Finally, we assessed Shared Reality Need (SR-N; four items; ω = 0.842, Cornwell et al., unpublished). This scale utilizes items such as “Sometimes things don’t feel “real” unless I experience them with others.” on a seven-point scale from “Strongly disagree” to “Strongly agree.” Scales that were not available in German (NTS, SI, SR-N, Perceived Generalized Similarity) were translated with the same procedure as the SR-T items.

#### Analysis Plan

We conducted the CFA using R and the packages *lavaan, semTools*, and *metaSEM* (Rosseel, 2012; Cheung, 2015; Jorgensen et al., 2018b), utilizing robust maximum likelihood estimation (MLM) with Satorra and Bentler (2001) scaled χ^2^. We evaluated model fit using the most-widely accepted methods (Hu and Bentler, 1998, 1999; Schermelleh-Engel et al., 2003): The χ^2^-test which should ideally not be significant; χ^2^ divided by degrees of freedom (χ^2^/*df*), which should be smaller than 3; the Comparative Fit Index (*CFI*) and the Tucker-Lewis Index (*TLI*), which should both be larger than 0.95, the root mean square error of approximation (*RMSEA*) and its 90% confidence interval and the standardized root means square residual (*SRMR*) which should both be smaller than 0.08.

To integrate the results across Studies 2 and 3, we used the approach described by Cheung and Chan (2005), which allows for a meta-analytical CFA across both samples. In Study 3, we additionally tested for measurement invariance across gender groups and conditions of partner credibility (Cheung and Rensvold, 2002; Milfont and Fischer, 2010): Comparing increasingly constrained models in a stepwise fashion to establish increasingly strict levels of invariance. First, we constrained factor loadings to be equal across measurement points to establish weak (or metric) invariance. Second, we additionally constrained intercepts to be equal, to test for strong (or scalar) invariance. Third, we tested strict invariance by comparing the scalar model to a model that also constrains residuals to be equal across tested groups. As recommended by Milfont and Fischer (2010), we evaluated model comparisons using the χ^2^-test as well as differences in *CFI* and gamma hat (*GH*; Steiger, 1989). χ^2^ should ideally not be significant, and Δ*CFI* and Δ*GH* should not be larger than 0.01 between models.

### Results

#### Item Descriptives

The descriptive statistics for the newly included Item 8 indicated normal distribution (Study 2: *M* = 3.366; *SD* = 1.446; *Skewness* = 0.136, *Kurtosis* = -0.956; Study 3: *M* = 4.109; *SD* = 1.386; *Skewness* = 0.175, *Kurtosis* = -0.036). The corrected item-total correlation with the other seven items in Study 2 was *r_it_* = 0.874, and 0.900 in Study 3. – the average *r_it_* being 0.833 in Study 2, and 0.885 in Study 3.

#### Factor Structure

We tested a five-item model corresponding to the one published in Schmalbach et al. (unpublished). Results of this analysis can be found in Table 6. The χ^2^ was significant and *RMSEA* was above the common cutoff criterion Study 1, but this fit index has been shown to be unreliable in models with high parsimony (Kenny et al., 2015). *CFI, TLI*, and *SRMR* evince excellent model fit in both studies and in the meta-analysis. Overall, the five-item model can, thus, be considered to be well-fitting. Internal consistency for the scale was high in both studies, ω_Study2_ = 0.941 [0.914; 0.959], ω_Study3_ = 0.959 [0.943; 0.975]. The excellent model fit in combination with the very high internal consistency and item-total correlations for the SR-T items, dissuaded us from testing any alternative models apart from the one-factorial solution. Additionally, we found evidence for strict measurement invariance across gender and partner credibility conditions (Table 7).

**Table 6:** Confirmatory factor analysis results (Studies 2 and 3).

Model	χ^2^ (*df*)^a^	*p*	χ^2^/*df*	*CFI*	*TLI*	*RMSEA* [90% *CI*]	*SRMR*
Study 2	13.406 (5)	0.020	2.681	0.983	0.966	0.123 [0.062; 0.185]	0.027
Study 3	7.904 (5)	0.162	1.581	0.995	0.990	0.055 [0.014; 0.087]	0.020
Meta-analysis	24.624 (5)	<0.001	4.925	0.996	0.991	0.114 [0.071; 0.160]	0.043

*^*a*^χ^*2*^ is Satorra-Bentler scaled.*

**Table 7:** Tests of measurement invariance across gender and partner credibility (Study 3).

Model	χ^2^ (*df*)^a^	Δχ^2^	Δ*df*	*p*	*CFI*	Δ*CFI*	*GH*	Δ*GH*
**Gender**								
Configural invariance	12.047 (10)				0.997		0.996	
Female	5.583 (5)				0.998		0.998	
Male	6.262 (5)				0.995		0.994	
Metric invariance	15.882 (14)	3.835	4	0.429	0.997	0.000	0.996	0.000
Scalar invariance	19.294 (18)	3.412	4	0.491	0.998	0.001	0.997	0.001
Strict invariance	19.751 (23)	0.457	5	0.994	1.000	0.002	1.007	0.010
**Partner credibility**								
Configural invariance	15.903 (10)				0.991		0.988	
High credibility	6.836 (5)				0.994		0.992	
Low credibility	9.915 (5)				0.984		0.980	
Metric invariance	20.287 (14)	4.384	4	0.357	0.990	0.001	0.987	0.001
Scalar invariance	24.410 (18)	4.123	4	0.390	0.990	0.000	0.987	0.000
Strict invariance	27.945 (23)	3.535	5	0.618	0.992	0.002	0.990	0.003

*^*a*^χ^*2*^ is Satorra-Bentler-scaled.*

#### Diagnostic Validity

In Study 2, there was a substantial correlation of the SR-T with recall tuning, *r*(110) = 0.355, *p* < 0.001, but not with message tuning, *r*(110) = 0.108, *p* = 0.258. We, then, controlled the SR-T’s prediction of recall tuning in a hierarchical regression analysis. Here we found evidence for the scale’s incremental validity over the IOS, the NTS, and Shared Identity, as SR-T remained the sole significant predictor of recall tuning, when accounting for the three in the hierarchical regression analysis (Table 8). In Study 3, there was a small significant association between SR-T and recall tuning *r*(190) = 0.159, *p* = 0.027. Analyses of message valence (Study 2) and recall valence (Studies 2 and 3) by experimental conditions are not of primary concern for the present study of the SR-T’s diagnostic validity and are presented in the Supplemental Material.

**Table 8:** Hierarchical regression of recall tuning on SR-T, controlling for IOS, SI, and NTS (Study 2).

		*B*	*SE*	β	*t*	*p*
1	***F*_(6,105)_ = 1.184, *p* = 0.321, *R*^2^ = 0.063, Adj. *R*^2^ = 0.010**					
	NTS self-esteem	0.007	0.103	0.008	0.064	0.949
	NTS belonging	-0.100	0.087	-0.122	-1.145	0.255
	NTS control	0.020	0.064	0.032	0.314	0.754
	NTS meaningful existence	-0.014	0.072	-0.026	-0.197	0.844
	Shared identity	0.212	0.110	0.201	1.936	0.056
	IOS	0.038	0.086	0.044	0.440	0.661
2	***F*_(7,104)_ = 2.732, *p* = 0.012, *R*^2^ = 0.155, Adj. *R*^2^ = 0.098**					
	**Δ*F*_(1,104)_ = 11.324, *p* = 0.001, Δ*R*^2^ = 0.092**					
	NTS self-esteem	0.030	0.099	0.039	0.304	0.762
	NTS belonging	-0.067	0.084	-0.082	-0.801	0.425
	NTS control	0.037	0.062	0.059	0.597	0.552
	NTS meaningful existence	-0.051	0.069	-0.094	-0.738	0.462
	Shared identity	0.123	0.108	0.116	1.137	0.258
	IOS	-0.029	0.085	-0.033	-0.338	0.736
	SR-T	0.282	0.084	0.339	3.365	0.001

*SR-T, shared reality about a target; NTS, need threat scales; IOS, inclusion of other in the self.*

#### Convergent/Discriminant Correlations

Associations between the SR-T and related measures are shown in Tables 9, 10. In Studies 2 and 3, as in Study 1, we again, found moderate to high associations with epistemic trust. Furthermore, the SR-T correlated moderately with all three measures of relational aspects (Relational Motivation, IOS, and SI). These associations were replicated in Study 3. Additionally, we found a small correlation with satisfaction of the need for control, and a moderate correlation with perceived generalized similarity – replicating findings by Schmalbach et al. (unpublished).

**Table 9:** Correlation matrix (Study 2).

	1	2	3	4	5	6	7	8	9	10	11	12	13	14
1 – SR-T	–	0.429***	0.312**	0.108	0.355***	0.007	-0.104	-0.188*	0.104	0.330***	0.095	0.116	0.076	0.317**
2 – Epistemic trust		–	0.352***	0.229*	0.143	0.004	-0.204*	-0.258**	0.117	0.202*	0.068	0.084	0.151	0.190*
3 – Relational motivation			–	0.078	0.095	0.117	-0.061	-0.101	0.266***	0.277**	0.101	0.061	-0.040	0.285**
4 – Message tuning				–	0.394***	-0.009	-0.095	-0.153	-0.009	0.004	0.123	0.132	-0.057	-0.006
5 – Recall tuning					–	-0.037	-0.137	-0.039	-0.070	0.209*	0.172	0.201*	-0.051	0.104
6 – NTS self-esteem						–	0.390***	0.179	0.660***	0.059	-0.058	-0.061	0.294**	0.037
7 – NTS belonging							–	0.095	0.415***	-0.036	0.117	0.121	0.071	-0.083
8 – NTS control								–	0.013	-0.289**	-0.035	-0.005	-0.046	-0.061
9 – NTS meaningful existence									–	-0.012	0.037	0.002	0.250**	0.064
10 – Shared identity										–	0.082	0.078	0.129	0.269**
11 – NFCC PSP											–	0.961***	-0.138	-0.113
12 – NFCC PNS												–	-0.089	-0.102
13 – NFCC undecidedness													–	0.021
14 – IOS														–

*^∗^Significant at the 0.05 level; ^∗∗^Significant at the 0.01 level; ^∗∗∗^Significant at the 0.001 level. SR-T, shared reality about a target; NTS, need threat scales; NFCC, need for cognitive closure; PSP, preference for structure and predictability; PNS, personal need for structure; IOS, inclusion of other in the self.*

**Table 10:** Correlation matrix (Study 3).

	1	2	3	4	5	6	7
1 – SR-T	–	0.159*	0.603**	0.444***	0.312**	0.221**	0.036
2 – Recall tuning		–	0.092	0.011	-0.049	-0.074	-0.036
3 – Epistemic trust			–	0.495***	0.244***	0.284***	-0.053
4 – Relational motivation				–	0.486***	0.391***	0.134
5 – Perceived similarity					–	0.273***	0.207**
6 – IOS						–	0.137
7 – SR need							–

*^∗^Significant at the 0.05 level; ^∗∗^Significant at the 0.01 level; ^∗∗∗^Significant at the 0.001 level. SR-T, Shared reality about a target; IOS, inclusion of other in the self; SR Need, shared reality need.*

### Discussion

Studies 2 and 3 were conducted to find evidence for the German SR-T’s construct and convergent validity – specifically its predictive power with regard to the recall bias found in the saying-is-believing paradigm. Fit was very good for the five-item model in both samples, and we found evidence for strict measurement invariance across experimental conditions. The SR-T predicted evaluative recall bias in both studies. Moreover, we found the expected correlations between epistemic and relational measures and the SR-T. These two findings present crucial evidence for the instruments convergent validity.

## General Discussion

We conducted this series of studies with the aim of developing and validating a German version of the SR-T. In Study 1, we found evidence for a strong single factor of experienced commonality, distinct from motivational corollaries, specifically, epistemic trust and relational motivation. In previous research, those two were often confounded with the core of shared reality. Accordingly, we tested a one-factorial scale in CFA, which evinced good fit in Studies 2 and 3 and in a meta-analysis across the two studies. Reliability for the scale was excellent in all three studies. In addition, we tested for and could confirm strict measurement invariance across gender groups and conditions of partner credibility. This means, that participants – irrespective of gender and whether their partner was a credible source of information or not – understand the SR-T the same way, and that it measures the construct of experienced commonality the same way. This is important because it enables comparisons of SR-T scores between the tested groups. Taken together with findings from Schmalbach et al. (unpublished), who presented evidence for invariance across targets, this offers strong support for the SR-T’s applicability in between- as well as with ingroup comparisons.

Across all three studies, we found strong associations between the SR-T and shared reality’s motivational corollaries. Epistemic trust in one’s communication partner consistently exhibited the highest correlations with the SR-T – a relationship that is well-founded theoretically and empirically (Echterhoff and Higgins, 2017). Relational motivation and interpersonal connectedness had slightly lower correlations, but still exhibited a strong link with experienced commonality. Similarly, perceived similarity had a moderate association with the SR-T. This pattern of correlations illustrates how shared reality is deeply embedded in motivational processes. First, individuals are often motivated *a priori* – epistemically and/or relationally – to experience shared reality. Second, shared reality has motivational consequences – increasing epistemic trust, relational motivation and connectedness with the other person. The relative primacy of epistemic processes in the current study may be due to the nature of the design which investigated interactions between strangers and not a general trend. It seems plausible that relational concerns should be more prevalent in interaction between familiar individuals. Finally, the findings demonstrate the crucial role of experiencing a shared reality with another person in satisfying basic human needs and forming and maintaining interpersonal relationships (Rossignac-Milon and Higgins, 2018).

In Study 1, we found strong evidence for the SR-T’s diagnostic capabilities. Namely, the SR-T accounted for a majority of the variance created by our employed manipulation. This confirmed that the SR-T is, in fact, a measure of perceived common inner states, in other words: shared reality. Even when controlling for the other state measures included in Study 1, the SR-T still explained close to half of the variance, showing that it is distinct from existing tools in providing an assessment of acutely experienced commonality.

Studies 2 and 3 dealt with the recall bias inherent in reproducing a previously read message in the saying-is-believing paradigm (Higgins and Rholes, 1978). In Study 2, we found a substantial, moderately high correlation between SR-T and the extent to which participants tuned their recall to fit their communication partner’s attitude – even when controlling for similar measures. These findings fit nicely with results from Schmalbach et al. (unpublished), who also found that the SR-T has unique predictive power – concerning attitudinal alignment – above and beyond established interpersonal scales such as the IOS and others. In Study 3, we replicated the association between SR-T and recall bias. However, the effect was distinctly smaller. This is evidence that not all recall bias is necessarily based on an experience of common inner states.

In saying-is-believing, a significant amount of variation in recall can be attributed to the extent to which participants felt they shared an inner state with their partner. That is, following their communication and the tuning of their recall, they were on the same wavelength with regard to the target of communication and expressed this in the SR-T. This was also the case in Study 3, but to a smaller degree. Since participants did not communicate with their supposed study partner, the creation of a shared reality was hindered. This is in line with Echterhoff and Higgins (2017) epistemic process framework, which emphasizes the need for a message production for the creation of a shared reality. Furthermore, it fits well with findings by Echterhoff et al. (2008), who demonstrated the need for appropriate communication for the emergence of a recall bias. These findings also lend credence and justification to previous shared reality research since Higgins and Rholes (1978) that used the recall bias as a quantitative measure of shared reality. To our knowledge this is the first time that a clear correlation has been demonstrated between the individuals’ experience of a common inner state with their partner and the extent to which their recall was biased.

The SR-T provides shared reality research with a standardized, quantitative dependent measure which is independent of the constraints of the evaluative recall bias. This will allow a broader community of researchers to access this important theory. Our five-item measure can easily be applied in a variety of research designs in interpersonal or intergroup communication and interaction of any kind. The German version in particular could be of interest for research dealing with relations between East- and West-Germany and how individuals of those formerly separated parts of a now unified country interact with one another and establish shared realities and common world views.

There are some limitations to the present series of experiments. First, Study 1 did not include Item 8. Thus, we cannot report its factor loadings in EFA, and its descriptive statistics stem from Studies 2 and 3. However, this only represents a minor caveat, given the excellent fit we found for the five-item model in Studies 2 and 3. It can be assumed that – even with the inclusion of Item 8 – the results presented in Study 1 would be largely the same given the very high internal consistency of the SR-T. Second, since participant ethnicity was not recorded in Study 1, we cannot control for potential confounding with the group membership manipulation. However, because of large cell sizes, it seems unlikely that participant ethnicities should be distributed so unevenly.

Some of the scales used for validation purposes had no published German equivalent and the authors translated them for the purpose of the present study. Pending a proper validation, the results should thus be interpreted with caution.

Finally, given the restrictions of our present samples, we only tested measurement invariance for gender and commonality condition, not age, as is commonly done as well. There is little reason to doubt the SR-T’s invariance across age, but pending an analysis in a suitable sample, researchers should not take it for granted.

Extending previous work by Schmalbach et al., (unpublished), we established the psychometric qualities of the SR-T in a German sample. In addition, we present further evidence for the measure’s convergent validity. Specifically, we relate the SR-T to the evaluative recall bias, which has previously been used as a primary measure of shared reality. Overall, we recommend the questionnaire as a tool for interpersonal and intergroup research, specifically for questions dealing with social perception and judgments.

## Ethics Statement

This study was carried out in accordance with the recommendations of the German Psychological Society. The protocol was approved by the ethics committe of the University of Münster’s Department of Psychology (2016-40-BSch). For the lab Study, subjects gave written informed consent in accordance with the Declaration of Helsinki. In the online Studies, they could only participate in the experiment after giving their informed consent by acknowledging that they have read and understood the study information.

## Author Contributions

BS and GE designed the studies and supervised the execution. BS and LH collected the data and did the analysis. All authors contributed to the writing of the manuscript and approved the final draft of the manuscript.

## Conflict of Interest Statement

The authors declare that the research was conducted in the absence of any commercial or financial relationships that could be construed as a potential conflict of interest.

## Appendix

**Appendix A:** The SR-T, both, German and English versions.

Ich denke, dass X und ich bezüglich Y auf derselben Wellenlänge liegen.	I think that X and I are on the same wavelength with regard to Y.
Ich empfinde ähnlich wie X in Bezug auf Y.	I feel the same way about Y as X does.
Ich stimme mit Xs Standpunkt in Bezug auf Y überein.	I agree with X‘s point of view of Y.
X und ich sehen Y gleich.	X and I see Y in the same way.
Ich stimme mit X Wahrnehmungen von Y überein.	I agree with X‘s perception of Y.

*Note. For the English SR-T items, please refer to Schmalbach, Rossignac-Milon, Keller,Higgins, and Echterhoff (unpublished) and contact the corresponding author for the full reference.*

**Appendix B:** Translations of the remaining items.

German	English
X und ich haben die gleichen Gedanken und Gefühle über Y.	X and I have the same thoughts and feelings about Y.
Ich stimme mit Xs Einstellungen in Bezug auf Y überein.	I agree with X‘s attitudes with regard to Y.
Meinem Empfinden nach steht meine Sichtweise auf Y mit der von X in Einklang.	I feel that my view harmonizes with X’s view of Y.
Man kann sich auf X Eindruck von den Bildern verlassen.	One can rely on X’s impressions of Y.
X ist eine glaubwürdige Informationsquelle in Bezug auf Y.	X is a reliable source of information with regard to Y.
X ist jemand, dessen Urteil über Y man vertrauen kann.	X is someone whose judgment about Y one can trust.
Ich möchte mich gut verstehen mit X.	I want to get along with X.
Ich denke, dass X ein sympathischer Mensch ist.	I think X is a likable person.
Ich würde gerne mehr Zeit mit X verbringen.	I would like to spend more time with X.

**Appendix C:** Translated description of Michael used in Study 3.

Michael works very independently. He tries to complete his tasks without asking any questions. He does so, even when he is unsure about details or when others’ advice could potentially improve his work.
He is an optimistic person. He loves to make jokes and to make light of problems and tension. Sometimes he doesn’t take important issues seriously.
Michael takes action and takes chances. He looks for opportunities to create successful research projects. He doesn’t spend much time considering potential risks and is not concerned about bending the rules.
Michael is very individualist and non-conforming. He holds strong opinions and ideas about how things should be done and sometimes he finds it difficult to restrain himself.
He is quite self-confident. He has a strong belief in his own abilities and gifts. This can sometimes come off as being arrogant and self-centered. In general, he is interested in his fellow students and tries to maintain friendships, but sometimes his strong personal focus puts people off.
