# Corrigendum: A Tool for Assessing the Experience of Shared Reality: Validation of the German SR-T

**DOI:** 10.3389/fpsyg.2019.01956

**Published:** 2019-08-30

**Authors:** Bjarne Schmalbach, Linda Hennemuth, Gerald Echterhoff

**Affiliations:** Department of Psychology, University of Münster, Münster, Germany

**Keywords:** shared reality, experienced commonality, common ground, scale development, communication, interpersonal relationships

In the original article, we neglected to include the funder “Alexander von Humboldt Foundation, DEU1072030/USA1163502,” to E. Tory Higgins (upon nomination by Gerald Echterhoff).

Furthermore, the manuscript “Schmalbach et al., unpublished” should be mentioned as “Schmalbach, Rossignac-Milon, Keller, Higgins, and Echterhoff, unpublished” in the Abstract.

A correction has been made to the **Abstract**:

“Humans are highly motivated to achieve shared reality – common inner states (i.e., judgments, opinions, attitudes) with others about a target object. Scholarly interest in the phenomenon has been rapidly growing over the last decade, culminating in the development of a five-item self-report scale for Shared Reality about a Target (SR-T; Schmalbach, Rossignac-Milon, Keller, Higgins, and Echterhoff, unpublished). The present study aims to validate the German version of the scale. Individuals can establish shared reality either by receiving social verification (i.e., agreement or confirmation from an interaction partner) or by aligning their inner state with that of their partner. To increase the scope of the present validation, we implemented both pathways of shared-reality creation in three studies (*N* = 522). Study 1 employed a social judgment task, in which participants assessed ambiguous social situations and received confirming (vs. disconfirming) feedback from their partner. Studies 2 and 3 build on the saying-is-believing paradigm, in which participants align their own evaluation of the target with their partner's judgment. Based on an evaluatively ambiguous description, participants communicated about a target person and later recalled information about the target (Study 2). To further generalize the findings, message production was omitted from the paradigm in Study 3. Overall, the five-item model of the SR-T evinced good fit and reliability. In Study 1, the SR-T reflected experimentally induced differences in commonality of judgments– even when controlling for several related state measures, such as Inclusion of Other in the Self and Need Threat. In Studies 2 and 3, the SR-T predicted participants' evaluative recall bias, which is an established, indirect index of communicators' shared-reality creation. This effect was stronger when participants overtly communicated with their study partner, but it still emerged without overt communication. Across all studies, correlations with related constructs support the convergent validity of the SR-T. In sum, we recommend the use of the SR-T in research on interpersonal processes and communication.”

Second, a note indicating the origin of the English SR-T items has been added. A correction has been made to **Appendix A**:

“*Note*. For the English SR-T items, please refer to Schmalbach, Rossignac-Milon, Keller, Higgins, and Echterhoff (unpublished) and contact the corresponding author for the full reference.”

Lastly, a correction has been made to the **Acknowledgments** section and should read:

“We thank Maya Rossignac-Milon, Victor Keller, and E. Tory Higgins for their contributions to the construction of the original, English SR-T scale.

The preparation of this article was facilitated by an Anneliese Maier Research Award from the Alexander von Humboldt Foundation, which has been awarded to E. Tory Higgins upon nomination by GE.

We acknowledge support from the Open Access Publication Fund of the University of Muenster.”

The authors apologize for these errors and state that they do not change the scientific conclusions of the article in any way. The original article has been updated.

