# Supplementary material for: A Tool for Assessing the Experience of Shared Reality: Validation of the German SR-T
Source: Front Psychol. 2019 Apr 16;10:832. doi: 10.3389/fpsyg.2019.00832 (PMC6478012; doi:10.3389/fpsyg.2019.00832)
Supplement: Supplementary file 1 [file Data_Sheet_1.docx]

**Supplemental Material**

Supplemental Material A
*ANOVA of message and recall bias and SR-T (Study 2)*

We found no effect of audience attitude on either, Message Valence, *F*(1, 108) = 1.826, *p* = .179, η²*_p_* = .017 [.000; .074], and Recall Valence, *F*(1, 108) = 0.207, *p* = .650, η²*_p_* = .002 [.000; .036]. Feedback about the communication’s effectiveness had a significant main effect on the message, *F*(1, 108) = 22.252, *p* < .001, η²*_p_* = .171 [.073; .270], but not on the recall, *F*(1, 108) = 0.045, *p* = .833, η²*_p_* = .000 [.000; .021]. The interaction effects were non-significant for both DVs, Message: *F*(1, 108) = 1.629, *p* = .205, η²*_p_* = .015 [.000; .071]; Recall: *F*(1, 108) = 0.004, *p* = .953, η²*_p_* = .000 [.000; .000]. Accordingly, the main effect of feedback, on the SR-T was not significant either, *F*(1, 108) = 2.217, *p* = .139, η²*_p_* = .020 [.000; .081]. As expected audience attitude and the interaction did have no significant effect on SR-T, *F* < 1, *p* > .500, η²*_p_* < .010.

Table A1

*Message valence by audience attitude and feedback condition (Study 2)*

|  | Success feedback | | |  | |  | | Failure feedback | | |
| --- | --- | --- | --- | --- | --- | --- | --- | --- | --- | --- |
|  | *M* | *SD* | *n* | |  | | *M* | | *SD* | *n* |
| Positive audience attitude | 0.558 | 1.037 | 30 | |  | | 1.442 | | 1.366 | 26 |
| Negative audience attitude | -0.117 | 1.524 | 30 | |  | | 1.423 | | 1.461 | 26 |

Table A2

*Recall valence by audience attitude and feedback condition (Study 2)*

|  | Success feedback | | |  | |  | | Failure feedback | | |
| --- | --- | --- | --- | --- | --- | --- | --- | --- | --- | --- |
|  | *M* | *SD* | *n* | |  | | *M* | | *SD* | *n* |
| Positive audience attitude | 0.317 | 0.846 | 30 | |  | | 0.346 | | 0.998 | 26 |
| Negative audience attitude | 0.217 | 0.989 | 30 | |  | | 0.269 | | 1.259 | 26 |

Table A3

*SR-T by audience attitude and feedback condition (Study 2)*

|  | Success feedback | | |  | |  | | Failure feedback | | |
| --- | --- | --- | --- | --- | --- | --- | --- | --- | --- | --- |
|  | *M* | *SD* | *n* | |  | | *M* | | *SD* | *n* |
| Positive audience attitude | 3.313 | 1.221 | 30 | |  | | 3.092 | | 1.127 | 26 |
| Negative audience attitude | 3.593 | 1.301 | 30 | |  | | 3.100 | | 1.400 | 26 |

Supplemental Material B
*ANOVA of recall bias and SR-T (Study 3)*

Recall valence was influenced significantly by the partner’s credibility status, *F*(1, 186) = 6.886, *p* = .009, η²*_p_* = .036 [.005; .087]. In contrast, the epistemic needs manipulation: *F*(2, 186) = 1.331, *p* = .267, η²*_p_* = .014 [.000; .046], and the interaction *F*(2, 186) = 1.599, *p* = .205, η²*_p_* = .017 [.000; .051], had no effect. Furthermore, there were no significant effects on SR-T by either Partner Credibility, *F*(1, 186) = 0.199, *p* = .656, η²*_p_* = .001 [.000; .021], the Epistemic Needs Manipulation, *F*(2, 186) = 0.608, *p* = .545, η²*_p_* = .006 [.000; .030], or the interaction *F*(2, 186) = 0.784, *p* = .458, η²*_p_* = .008 [.000; .034]

Table B1

*Recall valence by epistemic needs and partner credibility (Study 3)*

|  | High credibility | | |  | |  | | Low credibility | | |
| --- | --- | --- | --- | --- | --- | --- | --- | --- | --- | --- |
|  | *M* | *SD* | *n* | |  | | *M* | | *SD* | *n* |
| High needs | 0.803 | 1.980 | 33 | |  | | 1.723 | | 1.270 | 37 |
| Control | 0.825 | 1.893 | 30 | |  | | 0.845 | | 2.347 | 29 |
| Low needs | 0.766 | 2.071 | 32 | |  | | 1.887 | | 1.060 | 31 |

Table B2

*SR-T by epistemic needs and partner credibility (Study 3)*

|  | High credibility | | |  | |  | | Low credibility | | |
| --- | --- | --- | --- | --- | --- | --- | --- | --- | --- | --- |
|  | *M* | *SD* | *n* | |  | | *M* | | *SD* | *n* |
| High needs | 3.812 | 1.336 | 33 | |  | | 4.151 | | 1.261 | 37 |
| Control | 3.960 | 1.262 | 30 | |  | | 4.103 | | 1.455 | 29 |
| Low needs | 4.338 | 1.498 | 32 | |  | | 4.110 | | 1.047 | 31 |
